# Demographics of the European Apicultural Industry

**DOI:** 10.1371/journal.pone.0079018

**Published:** 2013-11-13

**Authors:** Marie-Pierre Chauzat, Laura Cauquil, Lise Roy, Stéphanie Franco, Pascal Hendrikx, Magali Ribière-Chabert

**Affiliations:** 1 Honeybee Pathology Unit, French Agency for Food, Environmental and Occupational Health & Safety (ANSES), European Union and National Reference Laboratory for honeybee health, Sophia Antipolis, France; 2 Unit of Epidemiology, French Agency for Food, Environmental and Occupational Health & Safety (ANSES), Lyon, France; 3 Epidemiological Surveillance Unit, French Agency for Food, Environmental and Occupational Health & Safety (ANSES), Scientific Affairs Department for Laboratories, Maisons-Alfort, France; University of Maryland, United States of America

## Abstract

Over the last few years, many European and North American countries have reported a high rate of disorders (mortality, dwindling and disappearance) affecting honeybee colonies (*Apis mellifera*). Although beekeeping has become an increasingly professional activity in recent years, the beekeeping industry remains poorly documented in Europe. The European Union Reference Laboratory for Honeybee Health sent a detailed questionnaire to each Member State, in addition to Kosovo and Norway, to determine the demographics and state of their beekeeping industries. Based on data supplied by the National Reference Laboratory for honeybee diseases in each European country, a European database was created to describe the beekeeping industry including the number and types of beekeepers, operation size, industry production, and health (notifiable diseases, mortalities). The total number of beekeepers in Europe was estimated at 620 000. European honey production was evaluated at around 220 000 tons in 2010. The price of honey varied from 1.5 to 40 €/kg depending on the country and on the distribution network. The estimated colony winter mortality varied from 7 to 28% depending on the country and the origin of the data (institutional survey or beekeeping associations). This survey documents the high heterogeneity of the apicultural industry within the European Union. The high proportion of non-professional beekeepers and the small mean number of colonies per beekeeper were the only common characteristics at European level. The tremendous variation in European apicultural industries has implication for any comprehensive epidemiological or economic analysis of the industry. This variability needs to be taken into account for such analysis as well as for future policy development. The industry would be served if beekeeping registration was uniformly implemented across member states. Better information on the package bee and queen production would help in understanding the ability of the industry to replace lost honey bee stocks.

## Introduction

In recent years, many European and North American countries have reported high rates of disorders (mortality, dwindling and disappearance) affecting their honeybee colonies (*Apis mellifera*) [1]–[3]. Honeybee colonies have been kept for the production of honey in Europe for thousands of years. Earlier beekeeping activities involved gathering honey from wild colonies. However, with the introduction of movable frame hives, more sophisticated management systems have developed. This has permitted the industry to become increasingly commercialized with the international production and sale of bee products including honey, royal jelly, packages (a box of worker bees used to set up a new colony), beeswax, propolis and others. These changes made the beekeeping an increasingly professionalized activity [4]. However, the demographics of the beekeeping industry in Europe is poorly described. The commercial figures that are available are provided by FAOSTAT (http://faostat.fao.org), the statistic division of the Food and Agriculture Organization (FAO), and Eurostat (http://epp.eurostat.ec.europa.eu), the statistical office of the European Union.

In recent decades, the poor health of honeybees has received considerable attention from the media and the public within the European Union [5], [6]. As honey bees are essential in the pollination of many agricultural crops, concerns have been raised about the ability of bees to maintain the pollination services required to ensure pollinator dependent food production [7]. Honeybees, alongside a multitude of other insects [8], [9], as well as some birds and even bats in other world areas, are part of the pollinator cohort. By transferring pollen from one plant to the another, pollinators facilitate the mixing plant genes which is crucial to maintaining sustainable biodiversity in wild ecosystems and agricultural land [10]. Various studies have estimated the economic value of pollination and the specific role of managed bees [11]. The production of 80% of the 264 crop species cultivated in the European Union depends directly on insect pollinators, and the global annual monetary value of pollination was estimated to be €153 billion [7]. The cost of the pollination service in wild ecosystems is even more difficult to estimate. Nevertheless, the pollination service provided by honeybees overall has a far greater economic significance than the sale of apicultural products alone.

There is a growing consensus that many factors contribute to the high rates of losses recently reported in Europe and in the United States [3], [12]. The degree to which these factors contribute to loss, either on their own or with other factors, is unknown. Epidemiological tools, developed to quantify risk factors in disease in other animal systems could greatly assist in improving our understanding of colony health. However, implementing such epidemiological studies requires knowledge of the targeted population (i.e. number of beekeepers and honeybee colonies), which is lacking for the European beekeeping industry. To address this lack of knowledge we initiated the current study in order to describe the European beekeeping industry by describing beekeeper demographics and honey bee health statistics. This information will in turn help facilitate future epidemiological studies designed at helping improve bee health.

## Materials and Methods

In order to protect and maintain the health status of bees, the European Commission designated the ANSES Sophia-Antipolis laboratory as the European Union Reference Laboratory for bee health (Honeybee EURL) on 29 October 2010 [13]. This decision was taken to follow up the communication from the European Commission on honeybee health [14] taking on board several concerns expressed, *inter alia*, in the report on “Bee Mortality and Bee Surveillance in Europe” [15]. The Honeybee EURL works with the National Reference Laboratory (NRL) of each member state of the European Union. In the case of honeybee health there is only one NRL per country. In 2011, the Honeybee EURL sent a questionnaire to the NRLs of the 27 European Union member states as well as contacts in Kosovo and Norway (Questionnaire S1). Survey contacts were asked to respond by a deadline of October 2011. A second questionnaire was sent in December 2011 with a request that responses be received by January 2012 (Questionnaire S2). Reminders were periodically sent to improve the participation of the European countries to the survey. All documents were written in English. The final answers were received late January 2012. The data presented in the present paper are from the responses of the NRLs for the year 2010. The survey aimed at collecting available information. It was not asked to the NRLs to conduct specific surveys to answer the Honeybee EURL request. All data communicated to the Honeybee EURL through this survey were already collected and available in the countries, whether this is the NRL point of view or the beekeepers’ one.

Data was collected through an ACCESS© database with 45 tables, 80 forms and 25 requests. Statistical processing of the data was achieved using basic descriptive methods (average ± standard errors).

## Results

### European Beekeeping Industry Figures

#### Population, distribution and density of honeybee colonies

Twenty five member states out of 27 as well as Kosovo and Norway responded to the survey. In 2010, the total number of colonies in Europe calculated from the questionnaires was 13 845 070. In Europe, the number of honeybee colonies per beekeeper was recorded in most of the countries (21 countries), but not in all of them. Therefore, this total number of honeybee colonies should be considered only as an underestimated estimation of the actual total.

The heterogeneity of colony distribution was relatively high in Europe. Five countries each had more than one million colonies (France, Greece, Italy, Poland and Spain) (Table 1), The country with the largest number of colonies in Europe being Spain (2 498 000 colonies, namely 18% of all European colonies). The data were transformed into relative numbers (number of colonies per km^2^), in order to compare colony density in the different European countries (Table 1). Colony density was also relatively heterogeneous in Europe (average 4.2±3 colonies/km^2^). Greece and Hungary had the highest density of colonies (about 10 colonies/km^2^). The lowest density (1 colony/km^2^ or less) was found in 6 countries located in the extreme north of Europe (Estonia, Finland, Ireland, Latvia, Norway and Sweden).

**Table 1:** Livestock (honeybee colonies), number of beekeepers, distribution and density of honeybee colonies in the European Union in 2010.

	No colonies(percentage of total)	No beekeepers(percentage of total)	Mean no colonies/beekeeper	Mean no colonies/km^2^
**Austria**	367 583 (2.7%)	24 453 (4.0%)	15.0	4.4
**Belgium**	110 000 (0.8%)	10 000 (1.6%)	11.0	3.6
**Bulgaria**	613 262 (4.4%)	27 477 (4.4%)	22.3	5.5
**Cyprus**	40 066 (0.3%)	**552 (0.1%)**	72.6	4.3
**Czech Republic**	517 300 (3.7%)	46 600 (7.5%)	11.1	6.6
**Denmark**	170 000 (1.2%)	5 000 (0.8%)	34.0	3.9
**Estonia**	42 000 (0.3%)	3 080 (0.5%)	13.6	1.0
**Finland**	37 500 (0.3%)	2 500 (0.4%)	15.0	0.1
**France**	1 346 575 (9.7%)	69 237 (11.2%)	19.5	2.5
**Germany**	680 000 (4.9%)	89 000 (14.4%)	7.6	1.9
**Greece**	1 500 000 (10.8%)	20 000 (3.2%)	75.0	**11.4**
**Hungary**	995 812 (7.2%)	17 556 (2.8%)	56.7	10.7
**Ireland**	**24 000 (0.2%)**	2 200 (0.4%)	10.9	0.3
**Italy**	1 127 000 (8.1%)	**70 000 (11.3%)**	16.1	3.7
**Kosovo**	70 664 (0.5%)	6 453 (1.0%)	11.0	6.5
**Latvia**	64 133 (0.5%)	3 500 (0.6%)	18.3	1.0
**Lithuania**	117 977 (0.9%)	4 565 (0.7%)	25.8	1.8
**Netherlands**	80 000 (0.6%)	8 000 (1.3%)	10.0	1.9
**Norway**	50 000 (0.4%)	3 000 (0.5%)	16.7	**0.1**
**Poland**	1 122 396 (8.1%)	44 951 (7.3%)	25.0	3.6
**Portugal**	580 065 (4.2%)	17 291 (2.8%)	33.6	6.3
**Romania**	963 342 (7.0%)	41 794 (6.8%)	23.1	4.0
**Slovakia**	246 214 (1.8%)	15 709 (2.5%)	15.7	5.0
**Slovenia**	156 178 (1.1%)	9 100 (1.5%)	17.2	7.7
**Spain**	**2 498 003 (18.0%)**	24 251 (3.9%)	**103.0**	4.9
**Sweden**	125 000 (0.9%)	12 000 (1.9%)	10.4	0.3
**United Kingdom**	200 000 (1.4%)	40 000 (6.5%)	**5.0**	1.3
**Europe**	13 845 070	618 269 (100%)	22.4	4.2

The minimum and the maximum are reported in bold in each column.

#### Number of beekeepers and size of the apiaries

In 2010, it was estimated that there were about 620 000 beekeepers in Europe (Table 1). Each beekeeper possessed an average of 22.4±23.7 colonies. The high standard error (23.7) illustrates the high heterogeneity in the number of colonies per beekeeper among the different European countries (Table 1). Spanish beekeepers had the highest average number of colonies per beekeeper (103.0) whereas British beekeepers owned the smallest average number of colonies (5.0).

Two percent (±3.1) of European operations (average across countries) possessed more than 300 colonies, 4±6.8% between 151 and 300 colonies, 16±19.8% between 51 and 150 colonies and the majority (78±25.2%) less than 50 colonies (Table 2). The overall tendency was toward small apiaries. The largest operations were observed in Greece, Italy and Romania.

**Table 2:** Description of the different types of beekeeper activities and sizes of the apiaries in Europe in 2010 (percentage).

	Operation size				Beekeeper activity			
Country	<50colonies	51–150colonies	151–300colonies	>300colonies	Professional	Non-professional	Part-time beekeepers	Hobby beekeepers
**Austria**	NS	NS	NS	NS	NS	NS	NS	NS
**Belgium**	NS	NS	NS	NS	1	99	0	99
**Bulgaria**	NS	NS	NS	NS	1.4	98.6	0	98.6
**Cyprus**	14	60	20	6	9	91	NS	NS
**Czech Republic**	93.8	6	0.2	0	0.2	99.8	15	85
**Denmark**	90	6	2	2	2	98	8	90
**Estonia**	75	20	4.9	0.1	1	99	29	70
**Finland**	90	NS	NS	0.4	4	96	14	82
**France**	93	4	2	1	3	97	7	90
**Germany**	98	NS	NS	NS	**0.1**	99.9	19.9	80
**Greece**	45	35	14.8	5.2	39.5	60.5	NS	NS
**Hungary**	20	73	5	2	7	93	73	20
**Ireland**	95	4	1	1	1	99	4	95
**Italy**	60	20	10	10	10	90	20	70
**Kosovo**	NS	NS	NS	NS	**88.4**	11.6	7.2	4.3
**Latvia**	83	14	2.5	0.5	3	97	14	83
**Lithuania**	NS	NS	NS	NS	3.1	96.9	NS	NS
**Netherlands**	95	5	1	0	1	99	5	94
**Norway**	94	5	1	0	1	99	17	82
**Poland**	90	9.5	0.4	0.1	0.5	99.5	NS	NS
**Portugal**	88	9	2	1	3	97	NS	NS
**Romania**	56.6	23.9	10.4	9.1	27	73	23.8	49.2
**Slovakia**	95	4	0.7	0.3	1	99	5	94
**Slovenia**	96.5	2.9	0.5	0.1	NS	NS	NS	NS
**Spain**	NS	NS	22.9	NS	22.9	70.5	NS	NS
**Sweden**	89	7	3	1	1	99	10	89
**United Kingdom**	96.5	1	1	1	1	99	NS	NS
**Europe**	78	16	4	2	**9.3**	**90.7**	**15.1**	**76.4**

NS: Not specified. The minimum and the maximum are reported in bold in each column.

### Beekeeping Activity

#### Description of the different types of beekeeping operations

In Europe, there was a common consensus amongst the countries that beekeeping activity could be categorized into hobby beekeepers, part-time beekeepers and professional beekeepers (Table 2). However, in the UK, Belgium, Bulgaria, Cyprus and Lithuania, no distinction was made between hobby and part-time beekeepers. In these countries, only two categories of beekeepers, professional and “non-professional” beekeepers were specified (“non-professional” beekeepers including both hobby and part-time beekeepers). Hobby beekeepers gain no income from their beekeeping activity. The definition of part-time beekeepers and professional beekeepers was dependent on the country. For part-time beekeepers, the beekeeping activity being not the main source of income was the only common trait across the countries (Table 3).

**Table 3:** Definition of professional beekeeper according to the different countries in Europe in 2010.

**Livestock size**	**>100 colonies**	-Finland
	**>150 colonies**	-Czech republic
		-France
		-Greece
		-Hungary
		-Latvia
		-Portugal
		-Spain
	**>200 colonies**	-Norway
	**>300 colonies**	-Sweden
	**>500 colonies**	-Romania
**Beekeeping is the main source of income**		-Denmark
		-Estonia
		-Germany
		-Ireland
		-Italy
		-Netherlands
**Others**		-Slovakia: legal form of business
		-United Kingdom: to be a member of the Bee farmers’ Association
**Not specified**		-Austria
		-Belgium
		-Bulgaria
		-Cyprus
		-Kosovo
		-Lithuania
		-Poland
		-Slovenia

In most of the European countries (11 countries), the definition of professional beekeepers depended on the number of colonies owned by the beekeeper, and was set at more than 150 colonies in 7 countries (Table 3). In 5 countries, the beekeeping activity had to represent the majority of the beekeeper’s income to qualify a beekeeper for “professional” status, regardless of the number of colonies involved. To summarize, the main variable used to define the professional beekeeper was either apiary size or source of income. For the rest of the paper, the beekeeper classification has not been changed from the original definitions provided by each European country. In other words, no standard definition was used when referring to professional beekeepers.

#### Distribution of the different types of beekeeping

**Figure 1:**
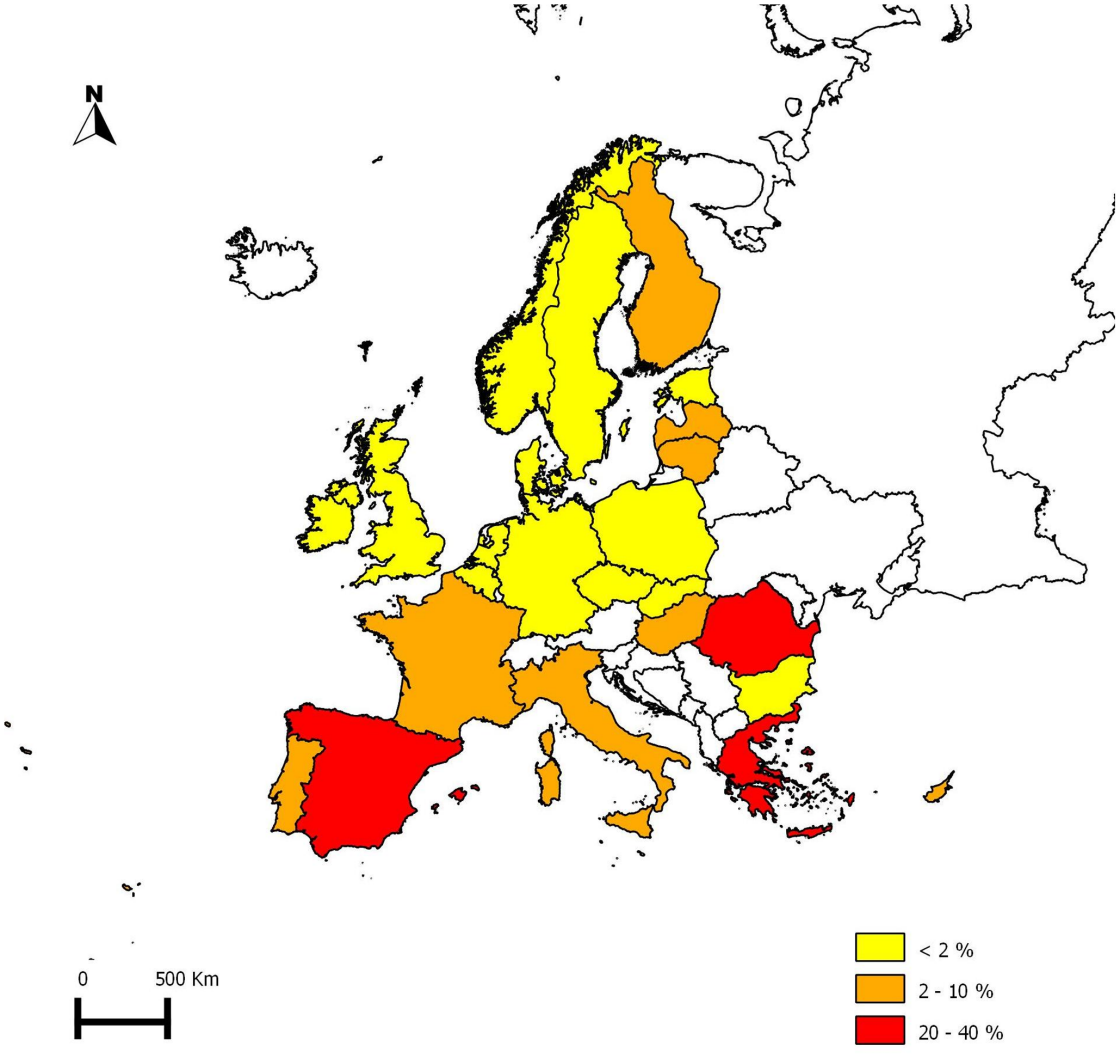
Percentage of professional beekeepers in Europe. Please see the text and Table 3 for the definition of “professional beekeepers”. Data are presented according to the definition given by each country.

In Europe in 2010, most of the beekeepers were hobby beekeepers (76.4±25.2%), whereas 9.3±19.1% were professionals (Table 2). However, in Kosovo and Hungary, 20% or less of beekeepers were hobbyists. In Romania, about half of all beekeepers were hobbyists. Except for Kosovo, Greece, Romania and Spain, professional beekeepers represented less than 10% of the total population of beekeepers (Figure 1, Table 2).

### Management of the Beekeeping Industry in Europe

There was no compulsory training for beekeepers prior to starting beekeeping activity in any of the EU countries. However, in Portugal and Romania some training was compulsory during beekeeping activity. In five countries (Portugal, Hungary, Romania, Slovakia and Spain), beekeepers needed to receive approval by the competent authority before starting the beekeeping activity.

Although an individual registration system existed for beekeepers in all European countries, with the exception of Austria, this identification was compulsory in only 20 countries. In Denmark, Kosovo, the Netherlands and the UK, beekeeper registration was voluntary. In Romania, registration was compulsory only for beekeepers who were members of a national program. In Ireland, registration was compulsory only for beekeepers with the intention of selling honey, in application of European Regulation (EC) No. 852/2004 on the hygiene of foodstuffs and (EC) No. 853/2004 on hygiene rules for food of animal origin. However, in Ireland and in most of the countries, many beekeepers claimed to be exempt from this requirement on the basis that they were only producing small quantities of honey.

In countries where registration existed, it was managed by various competent authorities to facilitate effective monitoring of the beekeeping industry (veterinarian services, food safety authorities, agricultural registers, animal production research centers and beekeepers’ associations). Fifteen countries had a centralized national database (Bulgaria, Cyprus, Czech Republic, Estonia, France, Germany, Hungary, Latvia, Lithuania, Portugal, Roumania, Slovakia, Slovenia and Spain), whereas 12 did not. Italy and Sweden intended to set up a central database in 2012. Furthermore, 21 countries recorded the geographical location of the beehives, and for 16 among them, recording the location was mandatory.

### European Honey Production

**Figure 2:**
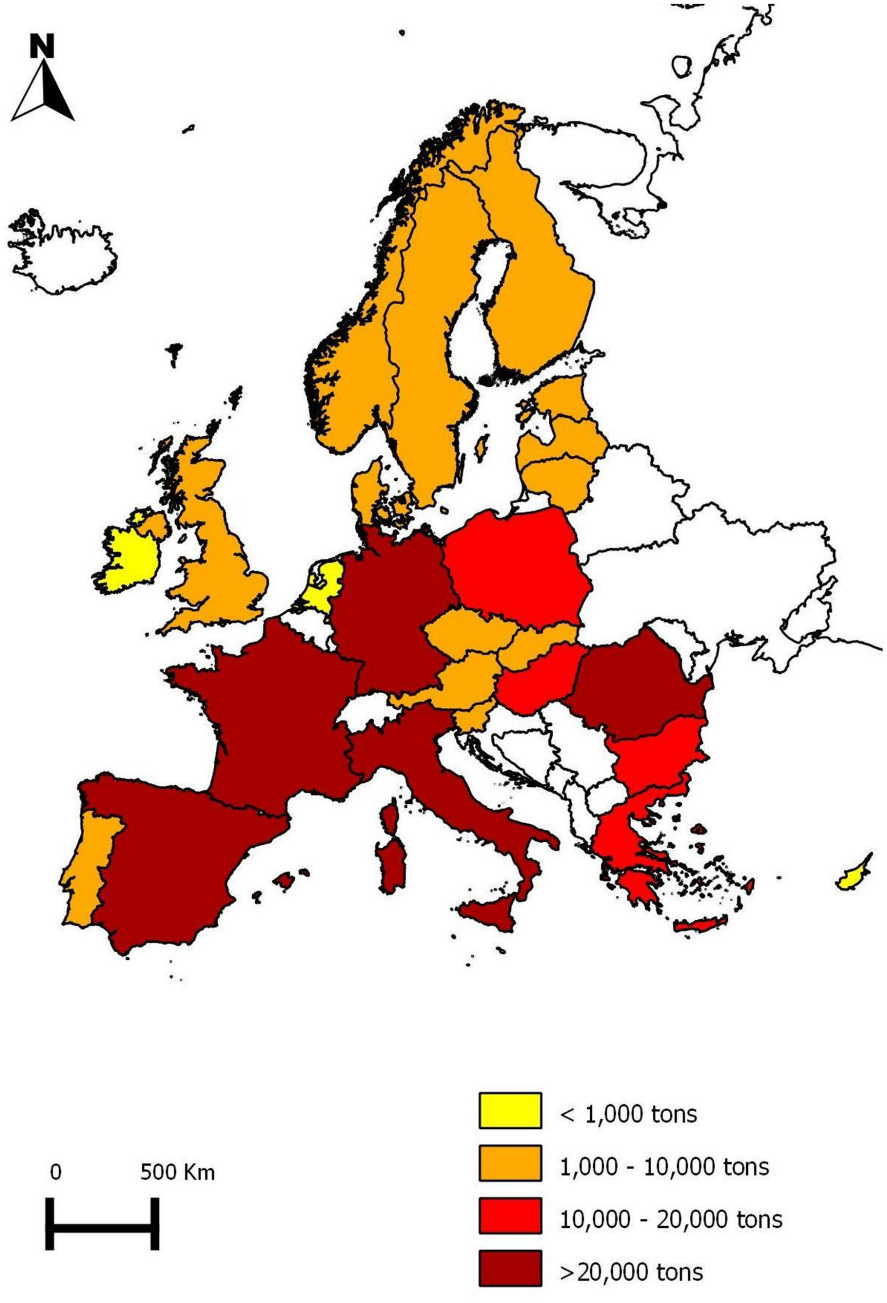
European honey production in 2010.

The main product of the beekeeping industry was honey. It was impossible to obtain data on the production of pollen, royal jelly, queens and packages at the European level (Table 4). In 2010, the total European honey production was estimated at over 220 000 tons, with average production of 4.8±4.5 tons/km^2^ and 1.6±0.8 tons/100 colonies (Table 4). Data were converted into relative numbers (tons of honey produced per 100 km^2^ and tons of honey produced per 100 colonies), in order to compare the density of production per country and colony productivity. Spain (33 000 tons) produced the most honey (Figure 2). When considering the amount of honey produced per 100 km^2^, Finland, Ireland and Norway were the countries with the lowest production (0.4 tons/100 km^2^), whereas Hungary was the country with the highest production per km^2^ (19.8 tons/100 km^2^). In terms of honey production per colony, the Netherlands had the least productive colonies (0.5 tons/100 colonies) while the Finnish colonies produced the highest quantity of honey (4.0 tons/100 colonies). The figures for productivity per area unit (100 km^2^) and per unit of production (100 colonies) show that European honey production was highly heterogeneous within the different countries in 2010.

**Table 4:** European production of bee products in 2010.

Country	Honey production (tons)	Honey production(tons/100 km^2^)	Honey production(tons/100 colonies)	Pollen production (kg)	Royal jellyproduction (kg)	Queen production(number)	Swarm production(number)
**Austria**	6 000	7.2	1.6	NS	NS	NS	NS
**Belgium**	NS	NS	NS	NS	NS	NS	NS
**Bulgaria**	10 595	9.5	1.7	NS	NS	55 000	10 000
**Cyprus**	590	6.4	1.5	893	7.5	1 000	NS
**Czech Republic**	7 455	9.5	1.4	NS	NS	60 000	NS
**Denmark**	3 000	7.0	1.8	500	5	20 000	2 000
**Estonia**	1 100	2.5	2.6	7 000	NS	NS	NS
**Finland**	1 500	0.4	**4**	1 000	NS	5 000	7 000
**France**	20 000	3.7	1.5	NS	7 000	NS	NS
**Germany**	20 441	5.7	3.0	NS	NS	NS	NS
**Greece**	15 000	11.4	1	NS	NS	NS	NS
**Hungary**	18 400	**19.8**	1.8	100 000	NS	45 000	1 000
**Ireland**	**250**	0.4	1.0	NS	NS	500	100
**Italy**	23 000	7.6	2.0	NS	4 000	350 000	NS
**Kosovo**	1 100	10.1	1.6	NS	NS	20 000	NS
**Latvia**	676	1.0	1.1	NS	NS	NS	NS
**Lithuania**	1 110	1.7	0.9	NS	NS	NS	NS
**Netherlands**	400	1.0	**0.5**	NS	NS	NS	ND
**Norway**	1 500	0.4	3	NS	NS	12 000	5 000
**Poland**	12 467	4.0	1.1	NS	NS	85 000	30 000
**Portugal**	7 426	8.1	1.3	NS	NS	NS	NS
**Romania**	22 224	9.3	2.3	100	3	40 000	85 000
**Slovakia**	3 160	6.4	1.3	100 000	30	75 000	50 000
**Slovenia**	1 700	8.4	1.1	NS	NS	26 000	NS
**Spain**	**33 084**	6.5	1.3	761 540	NS	NS	NS
**Sweden**	3 500	0.8	2.8	NS	NS	NS	NS
**United Kingdom**	6 000	4.0	3	NS	NS	4 500	5 000
**Europe**	**221 678**	**4.8**	**1.6**				

NS: Not specified. The minimum and the maximum are reported in bold in each column.

About 200 000 tons of honey were imported into the different countries of the community in 2010; while 90 000 tons were exported from the countries (Table 5). Germany imported and exported the largest quantities of honey in Europe in 2010 (respectively around 90 000 tons and 20 000 tons). The main honey distribution network was retail distribution. The price of honey varied from 2 to 40€/kg depending on the country and on the distribution network. The wholesale honey price ranged from 2 to 14€/kg and the retail honey price ranged from 3 to 40 €/kg.

**Table 5:** Trade of honey: quantity of honey imported into and exported from the European Union; volume of honey sold through retail or wholesale distribution and price range for honey in Europe in 2010.

Country	Imported honey (tons)	Exported honey (tons)	Honey retail distribution (tons)	Honey wholesale distribution (tons)	Retail price(€/kg)	Wholesale price(€/kg)
**Austria**	6 124	1 232			7–14	3–6
**Belgium**						
**Bulgaria**	230	8 540	2 944	3 637	**3**–8	**2**–3
**Cyprus**						
**Czech Republic**	2 172	1 144	5 000	2 000	3–5	**2**–3
**Denmark**	2 500	1 450	2 500	1 000	8–**40**	3–6
**Estonia**	**165**	0.4			4–6	6–7
**Finland**	1 286	**0.1**	2 700		4–8	
**France**	28 000	5 000	11 500	8 500	6–10	3–6
**Germany**	**89 550**	**20 529**			7–19	**2**–**14**
**Greece**	1 950	700			7–20	4–6
**Hungary**	700	14 400	4 000	15 000	5–8	3–4
**Ireland**	1 156	163	75	25	10–14	6–8
**Italy**	10 000	3 000			6–9	3–5
**Kosovo**					6–10	
**Latvia**	285	234	972	874	5–10	**2**–3
**Lithuania**	500					
**Netherlands**	8 500		75 000		8–14	5–10
**Norway**	250	10	750	750	10–25	4–5
**Poland**	11 621	2 721	10 426	2 041	4–10	3–6
**Portugal**	1 376	1 057				
**Romania**	880	11 017	10 000	10 000	4–5	
**Slovakia**	400	200	1 660	1 500	4–8	**2**–4
**Slovenia**	580	40	1 530	170	5–10	3–4
**Spain**	4 626	18 799	3 493	16 248	4–8	**2**–3
**Sweden**	3 000		2 100	1 200	6–9	5
**United Kingdom**	30 000	2 000	20 000	27 500	12–18	6–7
**Total**	**205 851**	**92 236.5**	**154 650**	**90 445**	**3–40**	**2–14**

The minimum and the maximum are reported in bold in each column.

### Honey Bee Health Status

#### Notifiable diseases, pests and pathogens

A notifiable disease has to be reported by law to the relevant government authorities. In this paper we use the term notifiable disease to designate the disease American foulbrood caused by the spore forming bacteria *Paenibacillus larvae*, and also the presence of the following pests and parasite in hives: the small hive beetle (*Aethina tumida*) and *Tropilaelaps* spp. mites. The three diseases listed as notifiable at European level, were considered as mandatory notifiable diseases in 23 countries. The small hive beetle and *Tropilaelaps* mites were not notifiable in Kosovo. In 2010, 5000 analyses were carried out in the European Union to detect *A. tumida*, and more than 8500 analyses were conducted for the detection of *Tropilaelaps* mites.

Twenty countries added other diseases, pests and pathogens to the above list, falling under national legislation (Table 6): varroosis the disease caused by the mite *Varroa destructor*, with the observation of clinical symptoms (in 18 countries), European foulbrood (15 countries), acariosis (11 countries), nosemosis (9 countries), and brood mycosis without mentioning if it was the common disease chalkbrood caused by *Ascosphaera apis* or the rarely observed stonebrood caused by *Aspergillus* spp. (4 countries). Romania also mentioned virosis as a notifiable disease, without mentioning which virus(es) were targeted.

**Table 6:** Notifiable diseases according to national legislation of Member States in Europe in 2010.

	AFB	SHB	*Tropilaelaps*spp.	Varroosis	EFB	*Acarapis* *woodi*	Nosemosis	Broodmycosis	Virosis	Total no of notifiable diseases
**Austria**	Yes	Yes	Yes	Yes	No	No	No	No	No	**4**
**Belgium**	Yes	Yes	Yes	Yes	Yes	Yes	No	No	No	**6**
**Bulgaria**	Yes	Yes	Yes	Yes	Yes	No	Yes	No	No	**6**
**Cyprus**										
**Czech republic**	Yes	Yes	Yes	Yes	Yes	No	No	No	No	**5**
**Denmark**	Yes	Yes	Yes	Yes	Yes	Yes	No	Yes	No	**7**
**Estonia**	Yes	Yes	Yes	Yes	Yes	Yes	Yes	No	No	**7**
**Finland**	Yes	Yes	Yes	No	No	No	No	No	No	**3**
**France**	Yes	Yes	Yes	Yes	No	No	Yes	No	No	**5**
**Germany**	Yes	Yes	Yes	No	No	No	No	No	No	**3**
**Greece**	Yes	Yes	Yes	No	No	No	No	No	No	**3**
**Hungary**	Yes	Yes	Yes	Yes	Yes	Yes	No	No	No	**6**
**Ireland**	Yes	Yes	Yes	No	Yes	No	No	No	No	**4**
**Italy**	Yes	Yes	Yes	Yes	Yes	Yes	Yes	No	No	**7**
**Kosovo**	Yes	**No**	**No**	Yes	Yes	No	Yes	No	No	**4**
**Latvia**										
**Lithuania**	Yes	Yes	Yes	Yes	Yes	Yes	Yes	No	No	**7**
**Netherlands**										
**Norway**	Yes	Yes	Yes	Yes	Yes	Yes	Yes	Yes	No	**8**
**Poland**	Yes	Yes	Yes	Yes	Yes	Yes	No	No	No	**6**
**Portugal**	Yes	Yes	Yes	Yes	Yes	Yes	Yes	Yes (ascophaerosis)	No	**8**
**Romania**	Yes	Yes	Yes	Yes	Yes	Yes	Yes	Yes	Yes	**9**
**Slovakia**	Yes	Yes	Yes	Yes	No	No	No	No	No	**4**
**Slovenia**	Yes	Yes	Yes	No	No	No	No	No	No	**3**
**Spain**	Yes	Yes	Yes	Yes	No	No	No	No	No	**4**
**Sweden**	Yes	Yes	Yes	Yes	No	Yes	No	No	No	**5**
**United Kingdom**	Yes	Yes	Yes	No	Yes	No	No	No	No	**4**
**Total number of countries**	**24**	**23**	**23**	**18**	**15**	**11**	**9**	**4**	**1**	

#### Reported causes of mortality

**Figure 3:**
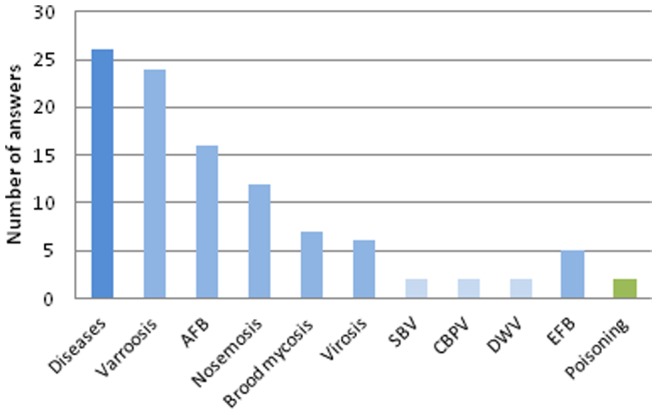
Main diseases observed in the field in Europe, in 2010. Diseases: non-specified diseases AFB: American foulbrood, SBV: Sacbrood virus, CBPV: Chronic bee paralysis virus, DWV: Deformed wing virus, EFB: European foulbrood.

**Figure 4:**
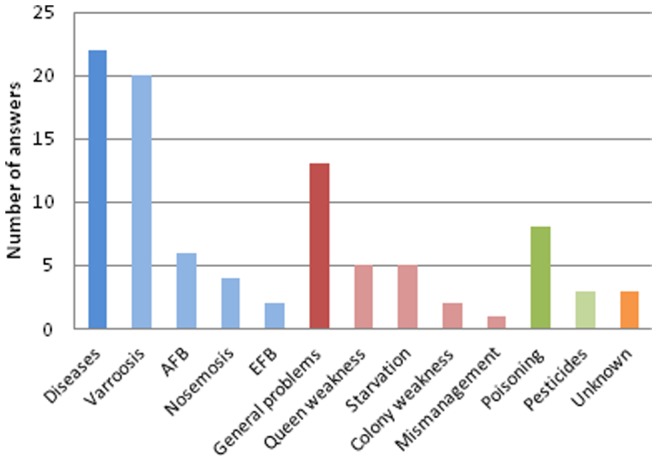
Main causes of colony mortality reported by beekeepers in 2010. Diseases: non-specified diseases, AFB: American foulbrood, EFB: European foulbrood.

**Figure 5:**
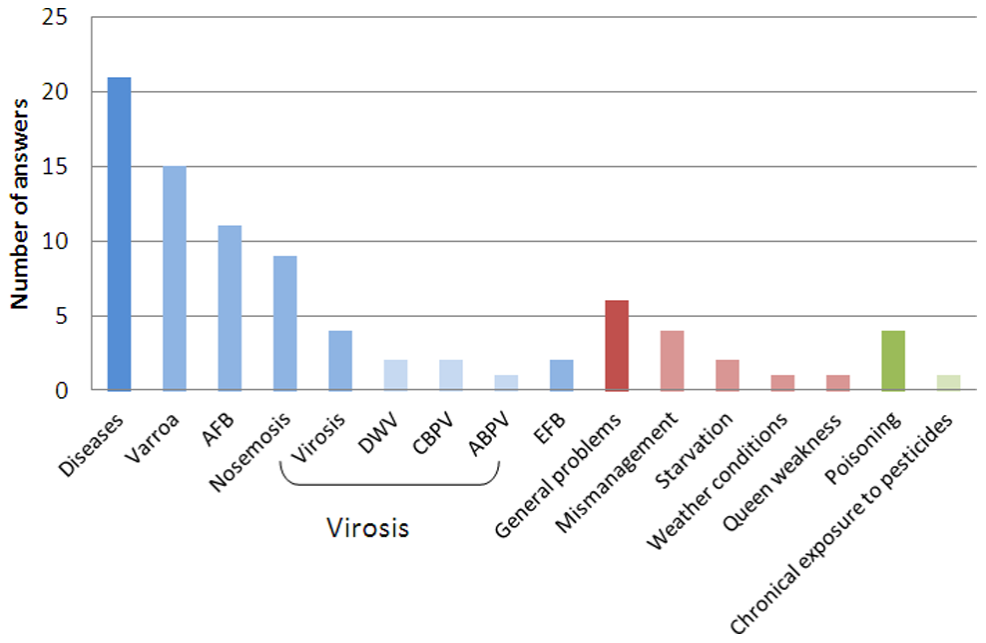
Main causes of colony mortality reported by the laboratories, in 2010. Diseases: non-specified diseases, AFB: American foulbrood, SBV: sacbrood virus, CBPV: Chronic Bee Paralysis Virus, DWV: Deformed Wing Virus, EFB: European foulbrood.

The questionnaire asked respondents to indicate the main diseases observed in the field and the causes of colony mortality reported by the beekeepers and by the laboratories. The figures reported were provided by the NRLs whether they originated from their own surveys or from beekeepers studies. The Honeybee EURL was interested in comparing the points of view from the NRLs and from the beekeepers. The data is all related to the year 2010. The data did not come from a monitoring program conducted by the Honeybey EURL. For the NRLs, varroosis was the most frequently reported disease from field observations in 24 questionnaires (Figure 3). It was also one of the main causes of mortality according to the beekeepers in 20 countries (Figure 4) and one of the main causes of mortality according to the NRLs in 15 countries (Figure 5). Apart from varroosis, the main diseases observed in the field were American foulbrood (in 16 countries), nosemosis (12 countries), brood mycosis (7 countries), virosis (6 countries) and European foulbrood (5 countries) (Figure 3). Two countries considered poisoning as the main problem observed in the field.

According to the beekeepers, the causes of mortality were multiple (Figure 4). Indeed, in 22 countries, the main reasons for colony losses were diseases (varroosis, American foulbrood, nosemosis and European foulbrood in decreasing frequency). In 13 countries, miscellaneous problems were at the origin of colony mortalities, sometimes listed by the NRLs as queen weakness, starvation, colony weakness and mismanagement. In 8 countries, according to the beekeepers, colony poisoning was a main cause of mortality, especially due to non-specified pesticides.

According to the laboratories, multiple factors were also at the origin of colony mortality. For 21 NRLs, the main reasons for colony losses were diseases: varroosis (15 NRLs), American foulbrood (11 NRLs), nosemosis (9 NRLs), and virosis (4 NRLs, including Deformed wing virus [DWV], Chronic bee paralysis virus [CBPV] and Acute bee paralysis virus [ABPV]). Miscellaneous problems were the main causes of mortality for 6 NRLs. Poisoning was the least frequent cause of colony mortality listed by the laboratories (4 NRLs). Only one laboratory mentioned chronic exposure to pesticides as an explanation for honeybee colony mortality (Figure 5).

The honeybee colony mortality rate was highly heterogeneous in Europe, ranging from a minimum of 7.5% in Slovakia (data provided by Coloss) to a maximum of 27.6% in Belgium (data from a local surveillance program). Rates differed according to the origin of the stakeholder (veterinarian services, local surveillance program, Coloss questionnaire (Coloss: prevention of honeybee COLony LOSSes was a worldwide network of bee researchers founded from 2008 to 2012 by a COST - European Cooperation in Science and Technology - action) or beekeepers’ association, Table 7). In some cases, colony mortality rates were not consistent within a country.

**Table 7:** Mortality of honeybee colonies in Europe in 2010, percentage and origin of the data.

	Colony mortality (%)
**Austria**	16.2^b^ –16.4^b^
**Belgium**	27.6^a^
**Denmark**	11^c^
**Estonia**	18.6^c^
**Finland**	13^c^–24^c^
**France**	20^b^–20^c^
**Germany**	9,2^a^ –16.3^a^
**Hungary**	30^d^–39^c^
**Ireland**	22^b^–17^b^
**Italy**	19^b^ –22.5^d^
**Lithuania**	20^c^
**Netherlands**	23^a^
**Norway**	9^c^
**Poland**	15^c^–17^b^
**Romania**	0.01^d^ –4.9^d^
**Slovakia**	7.5^b^–−15^c^
**Slovenia**	12^d^–23^c^
**Sweden**	24.5^c^
**United Kingdom**	12^d^–14^c^

a Local surveillance projects;

b Coloss questionnaire;

c beekeeper associations;

d veterinary services.

## Discussion

This study succeeded in gathering new information on beekeeping in Europe. The survey was a key step for setting up the European NRL network for honeybee health. Unlike diseases affecting other sources of food production, the NRLs on honeybee health have only recently begun to get organized in some countries of the European Union. The European Commission designated the EURL for bee health in 2010, and it began its activity in 2011 [13]. Some laboratories were only appointed as an NRL in 2012. Therefore, data currently generated from the different NRLs may not be comparable to each another. As a result, no reliable assessment of the colony health status is available to date at the European level.

From our results, it was clear that the beekeeping industry was highly heterogeneous from one European country to another in many areas (number of beekeepers, density of honeybee colonies, size of the apiaries, honey production). This could be due to historical traditions and climatic conditions. The climate in the northern countries was less suitable to high-production beekeeping than that of southern parts of Europe. Historical references support the major influence of climatic conditions on beekeeping development [16], [17]. The first evidences of beekeeping was found to be in the Mediterranean area. In 2010, 47% of the colonies of the European Union were still located in Southern Europe - France, Greece, Italy and Spain.

The Honeybee EURL activity is part of regulatory system applied to all animal species that produce food for humans. Therefore, the European Commission expects that the Honeybee EURL achieves tasks routinely implemented in other food productions industries. However, the beekeeping industry presented numerous particularities compared to other food productions. The size of the apiary was generally small (22.4 colonies/beekeeper). Unlike the cattle or pig industry, most beekeepers were still hobby beekeepers in 2010, sometimes referred to as “non-professional” beekeepers. The different terms used in each European country to designate beekeeping activity were also a cause a difficulty in comparing figures from different countries. The official definition of “professional beekeeper” was not the same in all European countries. However, in some official documents from the European Commission, the criterion used to classify a beekeeper as “professional” was the number of colonies owned, which should be more than 150 colonies [18], [19]. Based on the declarations from each country, the definition of “professional beekeeper” in the European countries was based on either a minimum number of colonies, or by income criteria, with beekeeping representing the main source of earnings. A standard definition of ‘professional beekeepers’ through Europe would allow comparisons between countries and the establishment of trends in time. The criterion of beekeeping representing the main source of earnings is the most relevant for establishing a unified definition of professional beekeepers at a European level, given that the minimum number of colonies required to ensure a living is highly variable from one country to another.

This survey also showed that it was impossible to obtain a complete set of data on European beekeeping. This was because of the high variability of colony registration requirements in the European Union. Indeed, in countries where colony registration was voluntary, the total population of beekeepers and colonies was consequently only an estimate. Even if colony registration was mandatory, registration of beekeepers and colony numbers was still not accurate in some countries. The requirement to officially declare colonies in order to be able to sell honey often deterred beekeepers from registering.

Considering the frequency of these non-declaration practices, it could be considered that the official figures on beekeepers and honeybee colony populations were underestimated at the European level. This underreporting made difficult to ensure correct health surveillance. As for other animal production sectors [20], information on the beekeeping industry should be based on the compulsory registration of each beekeeper and honeybee colony. This record should be managed by a competent authority, which would be in charge of a centralized national database in each European country. This would enable a rapid and efficient response by the health authorities in the event of a major health crisis [20] and eventually lead to a better understanding of honeybee health.

In animal diseases, transmission is highly dependent on host density [21], as has been shown in beekeeping with *V. destructor* [22], [23]. New infestations or re-infestations of honeybee colonies are facilitated if colony density is high [24]. As for other characteristics of beekeeping, colony density was quite variable from one country to another, with higher densities in Southern Europe than in Northern Europe. In the case of detection of one of the exotic pests targeted by the EU legislation (*A. tumida* or *Tropilaelaps* mites), the success of eradication is dependent on the availability of hosts (honeybee colonies) for the pest to feed and reproduce on [25]. Again, reliable figures on the number of honeybee colonies and their geographical locations are key factors required for effective control of honeybee diseases.

Global trade of honeybees and other goods has accelerated the spread of ‘new’ pathogens, predators and pests to other parts of the world [26]. The European Union is highly concerned by the risk of introduction of *A. tumida* and *Tropilaelaps* mites into Europe. In September 2004, two immature *A. tumida* larvae were found in cages of mated *Apis mellifera ligustica* queens and attendants imported from Texas (USA) to Portugal. All beehives of the apiary and another apiary 5 km from the first apiary were burned and the soil layer was removed and buried deep in the ground. The locations where beehives had been located were covered with plastic and the soil was flooded with permethrin [27]. Since this event, there have been no reports of detection of *A. tumida* in Portugal or elsewhere in Europe. In order to prevent the introduction of these pests, it is mandatory that each Member State report any suspicious cases [28]. Indeed, several thousands of prophylactic analyses were conducted in 2010 to ensure that the two pests were not present in Europe. Data on honey production provided by the NRLs were consistent with those provided by FAOSTAT for 2010. However, there was a surprising lack of information concerning the production of pollen, royal jelly, queens and swarms. When referring to beekeeping, these products are often considered to be secondary and information about them is often erroneous at the national level. Given the colony decline observed in many countries, the trade of swarms and queens is becoming economically significant. Therefore, statistics on their production would provide relevant indications on the need for the renewal of honeybee livestock in each European country.

The estimated causes of colony mortality differed depending on the different stakeholders that provided data, mainly because additional information is available in NRLs. The diseases claimed to be involved in colony losses by the beekeepers and the NRLs were the same (varroosis, nosemosis, American and European foulbrood). The NRLs mentioned additional diseases, caused by viruses (CBPV, DWV and ABPV). Thank to their analyses, NRLs were able to confirm symptoms by the detection and sometimes the quantification of the different pathogens. Moreover one of the main tasks of the EURL for bee health is to develop, validate and harmonise the use of standardised methods by the NRLs for diagnosis. The overall purpose is to ensure the consistency and efficiency of honey bee disease surveillance at the European level.

Beekeeping is more dependent on complex environmental factors than any other animal or food production industry. The main causes of colony mortality reported in Figures 3, 4, and 5 are strictly qualitative and subjective and therefore must be handled with caution. Pesticide poisonings, which have often captured the attention of media in the past, were only occasionally reported in the beekeepers statements. This is consistent with the results of several recent research studies [29]. Indeed, assessing the impacts of pesticides on honeybee health revealed to be highly complex and, in most cases, different from direct acute poisoning [30], [31]. Interactions between pesticides and pathogenic microorganisms result in an increased bee mortality as shown in laboratory experiments [32]–[34]. In the field, possible sublethal effects on honeybee colonies have been shown after the implementation of a rather technical protocol [35]. These methodological difficulties might lead to the underestimation of the actual deleterious impact of pesticides on honeybee health.

Rates of colony mortality fell, as expected, within a wide range of values (from 7.5 to 27.6%). These variations are likely to be linked to the high heterogeneity described above and to the protocols implemented to collect information. There were no standard protocols implemented at European level to collect epidemiologically sound information on honeybee colony losses. For some years, the Coloss network has distributed a questionnaire to a wide range of countries, including Europe, based on the beekeeper voluntary participation. The results have been included in this paper but are not directly comparable to other studies given the bias in sampling beekeeper populations (no data randomization was possible due to the voluntary participation of the panel [12], [36] although some local attempts have been achieved in Belgium for example [37]). In North America, honeybee colony losses were also recorded [38]. In the United States, the Bee Informed Partnership has performed a survey for several years using the same method of sampling (online survey of a convenience and snowball sample of beekeepers (for details see [39]–[41]), producing comparable results between years. Classical epidemiological methods have scarcely been used when studying honeybee losses. Recently, this field of work started to be adapted to understand and reduce honey bee mortality [42], [43]. In 2012, the European Commission, with the technical support of the Honeybee EURL initiated a pan-European surveillance study on colony losses. For the first time, colony mortality will be recorded in a consistent manner in 17 countries of the European Union through an epidemiologically sound protocol. Samples, field observations and beekeeping information will be collected and recorded according to a protocol uniformly applied in each country, to assess the prevalence of 7 diseases, pests or pathogens (varroosis, American foulbrood, European foulbrood, Nosemosis, DWV, ABPV, CBPV), to improve if needed the early detection of the two exotic arthropods, *Aethina tumida* and *Tropilaelaps* spp. mites, and to establish possible risk factors for colony losses [44].

## Conclusion

Thanks to the information provided by the NRLs, this study reports the results of a questionnaire on the beekeeping industry in Europe. Great differences between countries were observed in nearly all the reported data (number of beekeepers, density of colonies, rates of colony mortality). The high proportion of non-professional beekeepers and the small mean number of colonies per beekeeper were the only common characteristics at European level. Throughout Europe (and throughout the entire world), there are strong historical traditions of beekeeping. Consequently, bee production was - and still is - mainly run by non-professionals. Nevertheless, given the increasing global exchanges and the ensuing emerging threats that affect honeybees, the Honeybee EURL highly recommends that registration procedures for beekeepers and honeybee colonies be improved within each country. It is also necessary to improve reporting of package bees and queen production and trade. These statistics would give relevant indications on the need for renewal of honeybee livestock in each European country.

## Supporting Information

Questionnaire S1 First questionnaire sent to the European countries on the activities of the National reference Laboratories for honeybee health for 2010.
(DOCX)

Questionnaire S2 Complementary questionnaire sent to the European countries dedicated to beekeeping sector.
(DOCX)
