# Supplementary material for: Demographics of the European Apicultural Industry
Source: PLoS One. 2013 Nov 13;8(11):e79018. doi: 10.1371/journal.pone.0079018 (PMC3827320; doi:10.1371/journal.pone.0079018)
Supplement: Questionnaire S1 — First questionnaire sent to the European countries on the activities of the National reference Laboratories for honeybee health for 2010. (DOCX) [file pone.0079018.s001.docx]

**Network of National Reference Laboratories for Bee Health**

**Initial assessment**

1. Name of the laboratory:

- **Please return before 03 October 2011 to the following address: eurl.bee@anses.fr**
- **Please also send us, if it exists, the beekeeping analysis request form distributed by your laboratory.**

**Thank you in advance for this information.**

Address:

**Sophia-Antipolis Laboratory –** EURL for Bee Health – 25 July 2011

Tel:

Email:

Fax:

Website address:

Country:

2. Name of the laboratory manager:

3. Contact person for the NRL for Bee Health/ Bee diseases:

Name:

Position:

Tel:

Mobile phone:

Email:

4. Possible replacement:

Name:

Position:

Tel:

Mobile phone:

Email:

General data on the beekeeping industry in the country:

## 5. Beekeeping industry in the country:

Number of beekeepers:      ,

Number of hives:

**Identification and registration of hives (legal framework)**

Are the beehives subject to compulsory individual identification?

If so what are the method(s) used?

Is there a central national database containing the up-to-date number of beehives in the country?

Does an up-to date central national register exist on apiaries either as food establishments or as other entities?

**Location of the hives**

Is the actual location of the hives known by the competent authority at all times? (Yes/No)

If so by which mechanisms is this achieved?

**Beekeeping**

Is beekeeping an activity subjected to approval by the competent authority prior its start? (Yes/No)

Is beekeeping an activity subjected to compulsory training of the beekeeper:

Prior to the start of the activity (Yes/No)? If yes, please specify.

During the activity (Yes/No)? If so please specify.

Type of beekeeping (expressed in percentage):

Professionals:      %

Definition of professional beekeepers in your country:

Part-time beekeepers:      %

Hobby beekeepers:      %

Percentages of beekeepers in the following categories (total number of colonies per beekeeper operation)

- <50 colonies:      %
- 51-150 colonies:      %
- 151-300 colonies:      %
- >300 colonies:      %

## 6. National production for 2010

- - Honey (tons):
  - Pollen (kg):
  - Royal jelly (kg):
  - Queens (estimated number):
  - Swarms (estimated number):
  - Pollination services (€):
  - Others, specify:

## 7. Commercial issues

- - Export:
    - Honey (tons):
    - Pollen (kg):
    - Royal jelly (kg):
    - Queens (estimated number):
    - Swarms (estimated number):
    - Others, specify:
  - Import:
    - Honey (tons):
    - Pollen (kg):
    - Royal jelly (kg):
    - Queens (estimated number):
    - Swarms (estimated number):
    - Royal jelly (kg):
    - Others, specify:
  - Honey distribution networks:
    - Retail (tons):
    - Wholesale (tons):
    - Others, specify (tons):
  - Estimate of the selling price per kg of honey in Euros:
    - Retail: between       € and       €
    - Wholesale: between       € and       €

## 8. Organisation of the industry

- - - The five main beekeeping associations:

| Name | Number of members | % of professionals | % of amateurs | Other characteristics |
| --- | --- | --- | --- | --- |
|  |  |  |  |  |
|  |  |  |  |  |
|  |  |  |  |  |
|  |  |  |  |  |
|  |  |  |  |  |

regulation and health status in the country:

## 9. Current regulations in the country and epidemiological survey

- - Regulated diseases:

- - Recent epidemiological survey :
    - Yes Specify the type of surveillance: Active (Yes/No):

Passive (Yes/No):

Length of time:

Clinical (field) analysis (Yes/No):

Laboratory analysis (Yes/No):

- - - No

## 10. Health status

- - Main diseases noted in the field:
  - Main causes of mortality reported by the beekeepers:
  - Main causes of mortality reported by the laboratory:
  - Other comments :

General data on the laboratory

## 11. Fields of expertise:

Animal health

Food hygiene

Water quality

Air quality

Soil quality

Radioactivity

Ecotoxicology

Other(s): specify

12. Please provide the date(s) when your laboratory was recognised as the official NRL for honeybees in your country:

13. What mandates has your laboratory been given?

Bee diseases (date of issue for this mandate: **)**

Physico-chemical contaminants in bee matrices  (date of issue for this mandate: **)**

Others, specify:       (date of issue for this mandate: **)**

14. Are there other laboratories in your country that act as NRLs in the field of bee health?

Yes Number :

No

In the fields:

Bee diseases, specify: ………………

Physico-chemical contaminants in bee matrices

Others, specify:

Name of this/ these laboratory(ies):

Address:

**Sophia-Antipolis Laboratory –** EURL for Bee Health

Contact person:

Tel:

Email:

Fax:

Website address:

Address:

**Sophia-Antipolis Laboratory –** EURL for Bee Health

Contact person:

Tel:

Email:

Fax:

Website address:

Address:

**Sophia-Antipolis Laboratory –** EURL for Bee Health

Contact person:

Tel:

Email:

Fax:

Website address:

Address:

**Sophia-Antipolis Laboratory –** EURL for Bee Health

Contact person:

Tel:

Email:

Fax:

Website address:

Address:

**Sophia-Antipolis Laboratory –** EURL for Bee Health

Contact person:

Tel:

Email:

Fax:

Website address:

Address:

**Sophia-Antipolis Laboratory –** EURL for Bee Health

Contact person:

Tel:

Email:

Fax:

Website address:

## Accreditations and official approvals

15. Is your Institution (Abency) accredited?

Yes Specify the accreditation body:       Specify the accreditation number:

Specify the accreditation standard:

No

16. Is your laboratory accredited in the field of bee diseases?

Yes Specify the scope of the accreditation:

Varroosis: mite detection (Yes/No):

Varroosis: disease: (Yes/No):

Tracheal mite: (Yes/No):

Nosemosis: (Yes/No):

American foulbrood: (Yes/No):

European foulbrood (Yes/No):

Brood mycosis (Yes/No):

Small hive beetle (Yes/No):

Tropilaelaps mites: *T. clarae*(Yes/No):

Tropilaelaps mites: *T. mercedesae* (Yes/No):

Amoeba disease (Yes/No):

Viruses, which one(s):

Other (specify):

No

17. Is your laboratory accredited in the field of screening for physico-chemical contaminants in bee matrices?

Yes Specify the scope of the accreditation:

Honey: (Yes/No):

Syrup: (Yes/No):

Adult honeybees: (Yes/No):

Honeybee larvae(Yes/No):

Honeybee pupae (Yes/No):

Pollen pellets (Yes/No):

Beebread (Yes/No):

Beeswax: (Yes/No):

Royal Jelly (Yes/No):

Other (specify):

No

18. Is your laboratory recognised/accredited for *Aethina tumida* and *Tropilaelaps* spp. screening in connection with imports from third countries?

Yes  Recognised  Accredited

No

19. Are there other laboratories in your country recognised/accredited for *Aethina tumida* and *Tropilaelaps* spp. screening in connection with imports from third countries?

Yes Number :        Recognised  Accredited

No

20. Does your laboratory plan on obtaining accreditation soon in the field of bee diseases?

Yes

No

Specify for which analytical technique(s):

- In the field of honeybee diseases

Varroosis: mite detection (Yes/No):

Varroosis: disease: (Yes/No):

Tracheal mite: (Yes/No):

Nosemosis: (Yes/No):

American foulbrood: (Yes/No):

European foulbrood (Yes/No):

Brood mycosis (Yes/No):

Small hive beetle (Yes/No):

Tropilaelaps mites: *T. clarae*(Yes/No):

Tropilaelaps mites: *T. mercedesae* (Yes/No):

Amoeba disease (Yes/No):

Viruses, which one(s):

Other (specify):

- In the field of pesticide analysis

Honey: (Yes/No):

Syrup: (Yes/No):

Adult honeybees: (Yes/No):

Honeybee larvae(Yes/No):

Honeybee pupae (Yes/No):

Pollen pellets (Yes/No):

Beebread (Yes/No):

Beeswax: (Yes/No):

Royal Jelly (Yes/No):

Other (specify):

## General analytical capacity of the laboratory

21. Number of agents working in your laboratory:

22. Main analytical techniques used^[[1]](#footnote-1):^

Clinical diagnosis

Bacterioscopy

Culture of agents (bacterial, fungal, etc.)

Immunological methods

Qualitative molecular biology techniques (PCR, RT-PCR, etc.):

Quantitative molecular biology techniques (real-time PCR, etc.):

Biochemical assays, according to which procedure:

Others, specify:


23. What is the proportion of analyses conducted in the bee industry compared to the overall analytical volume of the laboratory?^[[2]](#footnote-2)^

**%**

Laboratory operations of the national reference laboratory in the field of honeybee health

## 24. Analytical capacity on bee diseases:

Analyses conducted:

| Disease/pathogen | Type of method used (principle, matrices), threshold of possible detection | Reference for the method(s)^[[3]](#footnote-3)^ | Number of analyses performed in 2010 | Mean analysis turnover^^[[4]](#footnote-4)^^ | Cost (excl. tax) of the invoiced analysis |
| --- | --- | --- | --- | --- | --- |
| Varroasis  Varroa destructor |  |  |  |  |  |
|  |  |  |  |  |  |
| Tracheal acariosis  Acarapis woodi |  |  |  |  |  |
|  |  |  |  |  |  |
| Nosemosis  Nosema apis /Nosema ceranae |  |  |  |  |  |
|  |  |  |  |  |  |
| American foulbrood  Paenibacillus larvae |  |  |  |  |  |
|  |  |  |  |  |  |
| Disease/pathogen | Type of method used (principle, matrices), threshold of possible detection | Reference for the method(s)^[[5]](#footnote-5)^ | Number of analyses performed in 2010 | Mean analysis turnover^^[[6]](#footnote-6)^^ | Cost (excl. tax) of the invoiced analysis |
| European foulbrood  Melissococcus plutonius |  |  |  |  |  |
|  |  |  |  |  |  |
| Brood mycoses  Ascosphaera apis, Aspergillus flavus |  |  |  |  |  |
| Asian hornet  Vespa velutina  (EC/DG envi) |  |  |  |  |  |
| Small hive beetle  Aethina tumida |  |  |  |  |  |
| *Tropilaelaps* spp.  *T. clareae*  *T. mercedesae*  Other, specify: |  |  |  |  |  |
| Diagnosis of viral diseases, specify:  Chronic bee paralysis (CBPV)  Sacbrood (SBV)  Other, specify: |  |  |  |  |  |
| Disease/pathogen | Type of method used (principle, matrices), threshold of possible detection | Reference for the method(s)^[[7]](#footnote-7)^ | Number of analyses performed in 2010 | Mean analysis turnover^^[[8]](#footnote-8)^^ | Cost (excl. tax) of the invoiced analysis |
| Viral detection, specify:  ABPV  AIV  BQCV  CBPV  DWV  FV  IAPV  KBV  SBV  SBPV  VDV  Other, specify: |  |  |  |  |  |
| Amoeba disease  Malpighamoeba mellificae |  |  |  |  |  |
| Other, specify: |  |  |  |  |  |
| Other, specify: |  |  |  |  |  |
| Other, specify: |  |  |  |  |  |
| Disease/pathogen | Type of method used (principle, matrices), threshold of possible detection | Reference for the method(s)^[[9]](#footnote-9)^ | Number of analyses performed in 2010 | Mean analysis turnover^^[[10]](#footnote-10)^^ | Cost (excl. tax) of the invoiced analysis |
| Other, specify: |  |  |  |  |  |
| Other, specify: |  |  |  |  |  |
| Other, specify: |  |  |  |  |  |
| Other, specify: |  |  |  |  |  |
| Other, specify: |  |  |  |  |  |
| Other, specify: |  |  |  |  |  |
| Other, specify: |  |  |  |  |  |

25. Technical facilities available for the pathological analysis of bees:

Premises available:


Equipment available:


Human resources:

Number of agents involved in the diagnosis of bee diseases in your laboratory:

Description of expertise of different agents involved:

| First and last name of the agent | Position^[[11]](#footnote-11)^ | Field of expertise^[[12]](#footnote-12)^ | Training carried out in the field of bee diagnosis and/or pathology | | |
| --- | --- | --- | --- | --- | --- |
|  |  |  | Title of the training course(s) attended | Training organisation(s) | Date |
|  |  |  |  |  |  |
|  |  |  |  |  |  |
|  |  |  |  |  |  |
|  |  |  |  |  |  |
|  |  |  |  |  |  |
|  |  |  |  |  |  |
|  |  |  |  |  |  |
|  |  |  |  |  |  |
| First and last name of the agent | Position^[[13]](#footnote-13)^ | Field of expertise^[[14]](#footnote-14)^ | Training carried out in the field of bee diagnosis and/or pathology | | |
|  |  |  | Title of the training course(s) attended | Training organisation(s) | Date |
|  |  |  |  |  |  |
|  |  |  |  |  |  |
|  |  |  |  |  |  |
|  |  |  |  |  |  |
|  |  |  |  |  |  |
|  |  |  |  |  |  |
|  |  |  |  |  |  |
|  |  |  |  |  |  |

## Culture collection/Laboratory sample library^[[15]](#footnote-15)^

26. Does your laboratory have a culture collection/laboratory sample library available for bee diseases?

Yes

No

27. If yes, specify which disease/pathogen the culture collection contains:

- American foulbrood  *Paenibacillus larvae*
- European foulbrood  *Melissococcus plutonius*  *Paenibacillus alvei*  Other pathogen, specify:
- Varroasis  *Varroa destructor*
- Acariosis  *Acarapis woodi*
- Nosemosis  *Nosema apis*  *Nosema ceranae*  Other, specify:
- Small hive beetle (*Aethina tumida*)
- *Tropilaelaps* *clareae*  *Tropilaelaps* *mercedesae*   Other species of *Tropilaelaps* , specify:
- Asian hornet *(Vespa velutina)*
- Amoeba disease *(Malpighamoeba mellificae)*
- Bee brood mycosis  *Ascosphaera apis*  *Aspergillus flavus*  Other pathogen*,* specify:
- Virus:
  - ABPV
  - AIV
  - BQCV
  - CBPV
  - DWV
  - FV
  - IAPV
  - KBV
  - SBV
  - SBPV
  - VDV
- Others, specify:

## Physico-chemical contaminants

28. Does your laboratory perform analyses in the field of physico-chemical contaminants in hive products?

Yes

No

If no, does another Institute perform the analysis for your laboratory?

Yes

No

If yes, please specify which institution:

In any case, specify below:

| Compound or class of compounds screened for | Type of method(s) used [Principle, matrices^[[16]](#footnote-16)^] | LOQ /Limit of quantification^^[[17]](#footnote-17)^^ | LOD  Limit of detection^^[[18]](#footnote-18)^^ | Reference for the method(s)^[[19]](#footnote-19)^ | Number of analyses performed in 2010 | Mean analysis turnover^^[[20]](#footnote-20)^^ | Cost (excl. tax) of the invoiced analysis |
| --- | --- | --- | --- | --- | --- | --- | --- |
|  |  |  |  |  |  |  |  |
|  |  |  |  |  |  |  |  |
|  |  |  |  |  |  |  |  |
|  |  |  |  |  |  |  |  |
|  |  |  |  |  |  |  |  |
|  |  |  |  |  |  |  |  |
|  |  |  |  |  |  |  |  |
|  |  |  |  |  |  |  |  |
| Compound or class of compounds screened for | Type of method(s) used [Principle, matrices^[[21]](#footnote-21)^] | LOQ /Limit of quantification^^[[22]](#footnote-22)^^ | LOD  Limit of detection^^[[23]](#footnote-23)^^ | Reference for the method(s)^[[24]](#footnote-24)^ | Number of analyses performed in 2010 | Mean analysis turnover^^[[25]](#footnote-25)^^ | Cost (excl. tax) of the invoiced analysis |
|  |  |  |  |  |  |  |  |
|  |  |  |  |  |  |  |  |

## Technical and scientific monitoring in the field of diagnoses of bee diseases

29. Does your laboratory perform a technical and scientific monitoring in the field of diagnoses of bee diseases (i.e. clinical symptom observation)?

Yes

No

30. If yes, according to which methods?

Specify which:


31. Does your laboratory perform extension services in the field of diagnoses of bee diseases?

Yes

No

32. If yes, according to which methods?

Specify which:


network of official laboratories in the country for honeybee diseases

## Coordination of the activities of official laboratories (network of laboratories) in the field of honeybee health

33. Is your NRL coordinate the activities of official laboratories responsible for the analysis (network of laboratories)?

Yes Number of laboratories :        Recognised  Accredited

No

Specify the scope of this network:

Bee diseases

Physico-chemical contaminants in bee matrices

Others, specify:

## 34. Does your laboratory organise training/information for network of laboratories

Yes

No

If yes, specify the frequency (number per year) of:

Training:

Information /meeting:

Specify on which disease(s)/ pathogen(s)


Specify on which pesticide(s)


## 35. Does your laboratory organise ILPTs (Inter-Laboratory Proficiency Tests)

Yes

No

If yes, specify frequency (number per year):

Specify on which disease(s)/ pathogen(s) the year of organisation and number of participants laboratories

- year:       number of participants laboratories :
- year:       number of participants laboratories :
- year:       number of participants laboratories :
- year:       number of participants laboratories :
- year:       number of participants laboratories :
- year:       number of participants laboratories :
- year:       number of participants laboratories :
- year:       number of participants laboratories :
- year:       number of participants laboratories :
- year:       number of participants laboratories :

Relationships in the beekeeping sector

## Relationships between the laboratory and other health stakeholders in the beekeeping sector

36. Who are the main users of your laboratory analytical services?

| Types of users | What proportion of analyses do they represent? (mean percentage) |
| --- | --- |
| Beekeepers |  |
| Veterinarians |  |
| Beekeeper groups, associations |  |
| Health authorities in your country |  |
| Research organisations, specify: |  |
| Others, specify: |  |
| Total | 100 % |

37. Is your laboratory involved or has it been involved in research or monitoring programmes specifically on bees?

| Objective  (epidemiological surveillance or research) | Description | Partners | Dates |
| --- | --- | --- | --- |
|  |  |  |  |
|  |  |  |  |
|  |  |  |  |
|  |  |  |  |
|  |  |  |  |
|  |  |  |  |
| Objective  (epidemiological surveillance or research) | Description | Partners | Dates |
|  |  |  |  |
|  |  |  |  |
|  |  |  |  |
|  |  |  |  |

38. Which publications, if any, is the laboratory involved with in the beekeeping sector? (the most relevant bibliographical references)


Expectations and needs

## 39. Diagnostic prospects

What are the analytical techniques/methods that you would like to develop in the field of bee disease diagnosis?


## Training/information

40. Would you like to receive support from the EU-RL within the framework?

Yes

No

41. If yes, according to what methods:

Participation in practical training sessions organised by the EU-RL

Specify in which sectors:


Technical and scientific exchanges during meetings or conferences

Specify the themes of interest:


Distribution of information on the subject (for example, by means of a mailing list)

## Inter-Laboratory Proficiency Tests (ILPTs)

42. Would you be interested in participating in an ILPT concerning an analysis method in bee pathology?

Yes

No

43. If yes, on which diagnostic method(s)? (list them in order of priority)


## 44. Comments and other proposals for activities to be implemented within the framework of the network of bee health laboratories

1. e.g.: Molecular biology techniques (PCR, RT-PCR, etc.), immunological methods, biochemical assays according to a particular method, etc. [↑](#footnote-ref-1)
2. Estimated % (estimated volume of bee analyses/total analytical volume of the laboratory) [↑](#footnote-ref-2)
3. Specify whether it is an OIE method, a method distributed by the NRL, an internal method (specify whether it is validated), etc. [↑](#footnote-ref-3)
4. Number of days between receipt of the sample and despatch of the results/opinion/interpretation [↑](#footnote-ref-4)
5. Specify whether it is an OIE method, a method distributed by the NRL, an internal method (specify whether it is validated), etc. [↑](#footnote-ref-5)
6. Number of days between receipt of the sample and despatch of the results/opinion/interpretation [↑](#footnote-ref-6)
7. Specify whether it is an OIE method, a method distributed by the NRL, an internal method (specify whether it is validated), etc. [↑](#footnote-ref-7)
8. Number of days between receipt of the sample and despatch of the results/opinion/interpretation [↑](#footnote-ref-8)
9. Specify whether it is an OIE method, a method distributed by the NRL, an internal method (specify whether it is validated), etc. [↑](#footnote-ref-9)
10. Number of days between receipt of the sample and despatch of the results/opinion/interpretation [↑](#footnote-ref-10)
11. e.g.: laboratory technician, veterinarian, etc. [↑](#footnote-ref-11)
12. e.g.: molecular biology, bacteriology, parasitology/entomology, toxicology, etc. [↑](#footnote-ref-12)
13. e.g.: laboratory technician, veterinarian, etc. [↑](#footnote-ref-13)
14. e.g.: molecular biology, bacteriology, parasitology/entomology, toxicology, etc. [↑](#footnote-ref-14)
15. e.g.: reference slides, nature of the preserved reference samples [↑](#footnote-ref-15)
16. e.g.: bees, bee bread, wax, honey, pollen, etc. [↑](#footnote-ref-16)
17. Lowest level of concentration at which the substance to be analysed can be quantified, in µg/kg (or ng/bee for the bee matrix). [↑](#footnote-ref-17)
18. Smallest quantity of a substance to be examined in a sample, which can be detected but not quantified as an exact value; in µg/kg (or ng/kg for the bees). [↑](#footnote-ref-18)
19. Specify whether it is an internal method (validated method or validation in progress). [↑](#footnote-ref-19)
20. Number of days between receipt of the sample and despatch of the results. [↑](#footnote-ref-20)
21. e.g.: bees, bee bread, wax, honey, pollen, etc. [↑](#footnote-ref-21)
22. Lowest level of concentration at which the substance to be analysed can be quantified, in µg/kg (or ng/bee for the bee matrix). [↑](#footnote-ref-22)
23. Smallest quantity of a substance to be examined in a sample, which can be detected but not quantified as an exact value; in µg/kg (or ng/kg for the bees). [↑](#footnote-ref-23)
24. Specify whether it is an internal method (validated method or validation in progress). [↑](#footnote-ref-24)
25. Number of days between receipt of the sample and despatch of the results. [↑](#footnote-ref-25)
