# Supplementary material for: Demographics of the European Apicultural Industry
Source: PLoS One. 2013 Nov 13;8(11):e79018. doi: 10.1371/journal.pone.0079018 (PMC3827320; doi:10.1371/journal.pone.0079018)
Supplement: Questionnaire S2 — Complementary questionnaire sent to the European countries dedicated to beekeeping sector. (DOCX) [file pone.0079018.s002.docx]

**Network of National Reference Laboratories for Bee Health**

**complementary questionnaire**

Name of the laboratory:

- **Please return before 30 January 2012 to the following address: eurl@anses.fr**

**Thank you in advance for this information.**

Country:

Name of the laboratory manager:

*Could you please complete again these few questions concerning general data on the beekeeping industry in your country. Indeed, due to a units imprecision in the answers, the data can’t be exploited. Thank you for your comprehension*

Identification and registration of hives

1. Is there an individual identification for each beekeeper? (Yes/No)
2. The beekeeper identification is:

Compulsory

Voluntary

Other (specify)

1. Is the total number of beehives recorded per beekeeper? (Yes/No)

If yes, how is it recorded?

With an individual number for each beehive

With the total number of hives per beekeeper

Other (specify)

1. How does the beekeeper register his hives?

By post

By remote contact (internet)

By phone

Other (specify)

1. Who is in charge of the registration?

Veterinarian services

Apiarist association

Other (specify)

1. Is there a central national database containing the up-to-date number of beehives in your country? (Yes/No)
2. Who is in charge/has access to this database?

Your National Reference Laboratory

A food safety agency

Other (specify)

1. Is there a control for these records?(Yes/No, if Yes specify)
2. Remarks:

location of the hives:

1. Are the beehives geographically localized? (Yes/No)
2. This geographical location is:

Compulsory

Voluntary

Other (specify)

1. Is there a record of the overwintering location? (Yes/No)
2. Is there a record of the beehive seasonal migration? (Yes/No)

If yes, how?

In an individual apiary book belonging to each beekeeper?

Through the declaration to the competent authority

Other (specify)

1. Is there a hive sanitary inspection before each hive movement? (Yes/No)

If yes, who is in charge of the inspection?

A veterinarian?

The beekeeper?

Is there an individual inspection of each hives?

Other (specify)

1. Remarks:

General data on the beekeeping industry in the country:

*TO COMPLETE AGAIN. BE CAREFUL OF THE UNITS.*

1. National production
   - Honey (**tons**):
   - Pollen (**kg**):
   - Royal jelly (**kg**):
   - Queens (**estimated number**):
   - Swarms (**estimated number**):
   - Pollination services (**€**):
   - Others, specify:
2. Commercial issues
   - Export:
     - Honey (**tons**):
     - Pollen (**kg**):
     - Royal jelly (**kg**):
     - Queens (**estimated number**):
     - Swarms (**estimated number**):
     - Others, specify:
   - Import:
     - Honey (**tons**):
     - Pollen (**kg**):
     - Royal jelly (**kg**):
     - Queens (**estimated** **number**):
     - Swarms (**estimated** **number**):
     - Others, specify:
   - Honey distribution networks:
     - Retail (tons):
     - Wholesale (tons):
     - Others, specify (tons):
   - Estimate of the selling price per kg of honey in Euros:
     - Retail: between       € and       €
     - Wholesale: between       € and       €
